# A scoping review on the impact of austerity on healthcare access in the European Union: rethinking austerity for the most vulnerable

**DOI:** 10.1186/s12939-022-01806-1

**Published:** 2023-01-05

**Authors:** Julia Nadine Doetsch, Clara Schlösser, Henrique Barros, David Shaw, Thomas Krafft, Eva Pilot

**Affiliations:** 1 EPIUnit – Instituto de Saúde Pública da Universidade do Porto, Porto, Portugal; 2 Laboratório para a Investigação Integrativa e Translacional em Saúde Populacional (ITR), Porto, Portugal; 3 Department of Health, Ethics & Society, Care and Public Health Research Institute, Faculty of Health, Medicine and Life Sciences, Maastricht University, Maastricht, the Netherlands; 4 Departamento de Ciências da Saúde Pública e Forenses e Educação Médica, Faculdade de Medicina, Universidade do Porto (FMUP), Porto, Portugal; 5 Institute for Biomedical Ethics, University of Basel, Basel, Switzerland

**Keywords:** Austerity, Healthcare access, Vulnerable populations, Unmet medical need, European Union

## Abstract

**Background:**

There is consensus that the 2008 financial and economic crisis and related austerity measures adversely impacted access to healthcare. In light of the growing debt caused by the COVID-19 crisis, it is uncertain whether a period of austerity will return.

**Objective:**

This study aims to provide a structured overview of the impact of austerity policies in the EU-28 zone, applied in response to the Great Recession, on access to health care for the adult population, using the five access dimensions by Levesque et al. (2013).

**Methods:**

This study followed the PRISMA extension for Scoping Reviews guideline. Medline (PubMed) and Web of Science were searched between February 2021 and June 2021. Primary studies in the English language published after the 1st of January 2008 reporting on the possible change in access to the healthcare system for the adult population induced by austerity in an EU28 country were included.

**Results:**

The final search strategy resulted in 525 articles, of which 75 studies were reviewed for full-text analysis, and a total of 21 studies were included. Results revealed that austerity policy has been primarily associated with a reduction in access to healthcare, described through four main categories: i) Increase in rates of reported unmet needs (86%); ii) Affordability (38%); iii) Appropriateness (38%); iv) and Availability and Accommodation (19%). Vulnerable populations were more affected by austerity measures than the general population when specific safeguards were not in place. The main affected adult vulnerable population groups were: patients with chronic diseases, elderly people, (undocumented) migrants, unemployed, economically inactive people and individuals with lower levels of education or socioeconomic status.

**Conclusion:**

Austerity measures have led to a deterioration in access to healthcare in the vast majority of the countries studied in the EU-28 zone. Findings should prompt policymakers to rethink the fiscal agenda across all policies in times of economic crisis and focus on the needs of the most vulnerable populations from the health perspective.

**Supplementary Information:**

The online version contains supplementary material available at 10.1186/s12939-022-01806-1.

## Introduction

After the first waves of the economic and financial crisis in the Eurozone between 2008 and 2010, also called the Great Recession, multiple European Union (EU) policymakers seeking to recover from the rise in deficits began to adopt austerity measures. These measures aimed to reduce overall government spending to lower national debt across Europe [1]. The call to invoke strong austerity measures was also supported by the European Commission (EC). Cooperating with the International Monetary Fund (IMF) and European Central Bank, the EC established the Troika, this newly created alliance agreed on a Memorandum of Understanding with multiple European member states [1]. The Memorandum of Understanding is a non-binding agreement between the parties. It defined the specific loan provided and the conditions and monitoring systems attached to it [1]. Starting in 2010, this monitoring mechanism imposed economic disciplinary regulations and certain austerity measures to be implemented in order to be eligible for bailout packages [2]. Hence, as a reaction to adverse financial circumstances, austerity emerged as the predominant policy response for multiple EU governments [3].

The Troika agreed with Cyprus, Greece, Ireland, and Portugal on specific economic adjustment programs that also included measures to reduce government spending in the healthcare sector to control deficits [4–7]. Besides the countries mentioned above, other countries such as Italy and Estonia also applied austerity measures to reduce public spending on healthcare, albeit at different levels, even though the Troika did not impose austerity upon these countries [8, 9]. These applied austerity measures can be understood as part of the predominant neoliberal policy scheme when placing it into a broader perspective. Neoliberal policy, besides referring to market-oriented reform policies (e.g., deregulating capital markets, lowering trade barriers), reduces the influence of the state in the economy by promoting austerity and privatisation [10].

Mladovsky (2012) categorised the different approaches on how austerity measures have been applied in the context of the healthcare sector [11]. EU countries’ policies were categorised into three major areas: (1) Measures that led to changes in the financing of health systems, such as the introduction of co-payments; (2) Measures that changed the scope of health services provided, such as Spain limiting its health services for migrants; (3) Measures that were intended to reduce the costs of publicly funded health care, such as wage cuts for healthcare workers [12]. An overview of all applied austerity measures can be found in Supplementary material 1.

Nevertheless, the times of minimising public spending on healthcare seem long ago amidst the current COVID-19 pandemic, where the primary governmental fiscal response was stimulus checks. However, in light of the new economic reality, some governments are advocating for new austerity measures, raising the possibility that many member states may enter a new era of austerity [13]. Even though it is reasonable to consider the economic effects of the pandemic, the policy of automatically creating relations between economic recessions and austerity should be reconsidered from a public health perspective. It is, therefore, of utmost importance to analyse the impact of past public policy practices on public health in the context of crisis events [14].

The underlying theoretical framework of the article is grounded in the “Political economy of health” (PEH) theory by Krieger (2001) [15] and the” Theory of Fundamental Causes” (TFC) by Link and Phelan (1995). The PEH theory distinguishes itself from psychosocial and ecosocial theories by emphasising the relationship between macro factors and health. The PEH theory focuses on “power relationships, government ideology and public policy, and welfare state typologies” (p.664) [17]. Whereas the TFC argues that “social factors such as socioeconomic status and social support are likely ‘fundamental causes’ of disease that, as they embody access to important resources, affect multiple disease outcomes through multiple mechanisms, and consequently maintain an association with the disease even when intervening mechanisms change” (p.80) [16]. Applying these two theories to this study results in the following hypothesis: Inevitably, health inequalities are substantially rooted in differences in access to resources, of which the latter is essentially the result of political and ideological decisions (As similarly argued by Szreter and Woolcock (2004) [18]).

This hypothesis is supported by the widespread agreement that the consequences of the 2008 financial and economic crisis and the subsequent austerity measures have adversely affected access to healthcare [19–22], defined as “the ability to reach and receive appropriate healthcare services in situations where there is a perceived need for care” [23]. Though a lot of studies have been conducted on the impact of austerity on health or healthcare in various EU countries, systematic overviews are scarce and/or address a particular scope (e.g., country, population group) [24]. The framework of Levesque et al. (2013) has been used before to assess healthcare access in multiple studies at the national level (for example Doetsch et al. (2017) [21]) and at the international level [25].

The present article distinguishes itself in its policy focus, addressing austerity, and provides cross-country comparison and evidence on the latter with a multi-national scale within the EU. To the best of the authors’ knowledge, this is the first study addressing the aforementioned subject in that dimension. Furthermore, its novelty lies in the patient-centric lens using the published framework of Levesque et al. (2013) to provide organised evidence.

Therefore, this study aims to provide a structured overview of the impact of austerity policies in the EU-28 zone, applied in response to the Great Recession, on access to health care for the adult population, using the five access dimensions by Levesque et al. (2013).

## Methods

The applied method is a scoping review which was chosen to map the body of literature on a topic area by providing an overview [26]. This study is reported according to the Preferred Reporting Items for Systematic Reviews and Meta-Analyses (PRISMA) extension for Scoping Reviews guideline (PRISMA-ScR) (Supplementary material 2). PRISMA is a 27-item checklist that is used to improve transparency in systematic reviews. The PRISMA-ScR, which is used in this article, is an extended version for scoping reviews which includes 20 main reporting items and 2 optional items to include when completing a scoping review. The main advantage is that it delivers a clear and comprehensive overview of available evidence on a given topic.

The methods section is organised according to PRISMA-ScR.

### Protocol and registration

Not applicable.

### Eligibility criteria

#### Definitions and specifications

Following Levesque et al. (2013), access to healthcare is defined as “the ability to reach and receive appropriate healthcare services in situations where there is a perceived need for care” [23]. The general definition includes characteristics on the demand side (healthcare users) and the supply side (healthcare providers). This study considered the supply side consisting of five main characteristics: approachability, acceptability, availability and accommodation, affordability, and appropriateness.

The definition of general unmet medical need (UMN) was taken from the EU-SILC survey, defined as a “Person’s own assessment of whether he or she needed examination or treatment for a specific type of healthcare, but did not have it or did not seek for it.” [27]. UMN can be seen as a proxy for measuring barriers in healthcare access [28, 29], as applied in the framework of Levesque et al. (2013) [23].

The EU-SILC survey primarily examines the UMN level; secondarily, it investigates respondents reported primary barrier to accessing healthcare (e.g., economic reasons, waiting lists, distance or lack of transport). These reasons are also called the UMN criterion [27]. This means that a decrease in one UMN criterion does not necessarily mean that this problem has been solved but that it could also be that another barrier just has become more imminent [30].

#### Population

The adult population was addressed.

#### Intervention

Studies must report austerity measures that may affect healthcare access on either “availability and accommodation”, “affordability”, or “appropriateness “[23]. The characteristics “approachability” and “acceptability”, concerned with transparency and out-reach and personal norms, were excluded as austerity measures do not directly affect them.

#### Setting

Studies needed to be conducted in an EU28 country. Studies should at least include one European country and report on the possible change in access to the healthcare system.

#### Study design

Only primary studies of quantitative and qualitative nature (case studies, longitudinal studies, cross-sectional studies) were included. Grey papers, reviews, commentaries, editorials, concept and opinion papers and other studies that were not formally published (e.g., conference abstracts) were not included due to the risk of reduced methodological quality and to avoid bias.

#### Comparator

Studies with or without a comparator group were included.

#### Other

Only publications written in English were considered. Studies must have been formally published and issued after the 2008 crisis (studies are being included from the 1st of January 2008).

### Information sources

Information sources: Two major databases, Primarily Medline (PubMed) as the most prominent database in the field of health-related publications and Web of Science, due to the economic and social scope, were consulted.

### Search

The research question was divided into three main topics: i) adults in European countries, ii) austerity, iii) and healthcare access (Supplementary material 3). After establishing the three main topics matching medical subject headings (MeSH terms) were searched for Pub Med and matched KeyWords Plus for Web of science. These broader terms were established by backwards-searching in the respective MeSH term library and KeyWords Plus library. Additional Keywords were added based on related synonyms and different spelling versions. For the topic “Europe”, all countries of the EU-28 were included as keywords. The search criteria for the general keywords were limited to the title and abstract for both databases. The final search strategy was entered into the advanced search form, linking the MeSH terms and keywords with the Boolean operator “OR” and the three main topics with the Boolean operator “AND”.

### Selection of sources of evidence

**Fig. 1:**
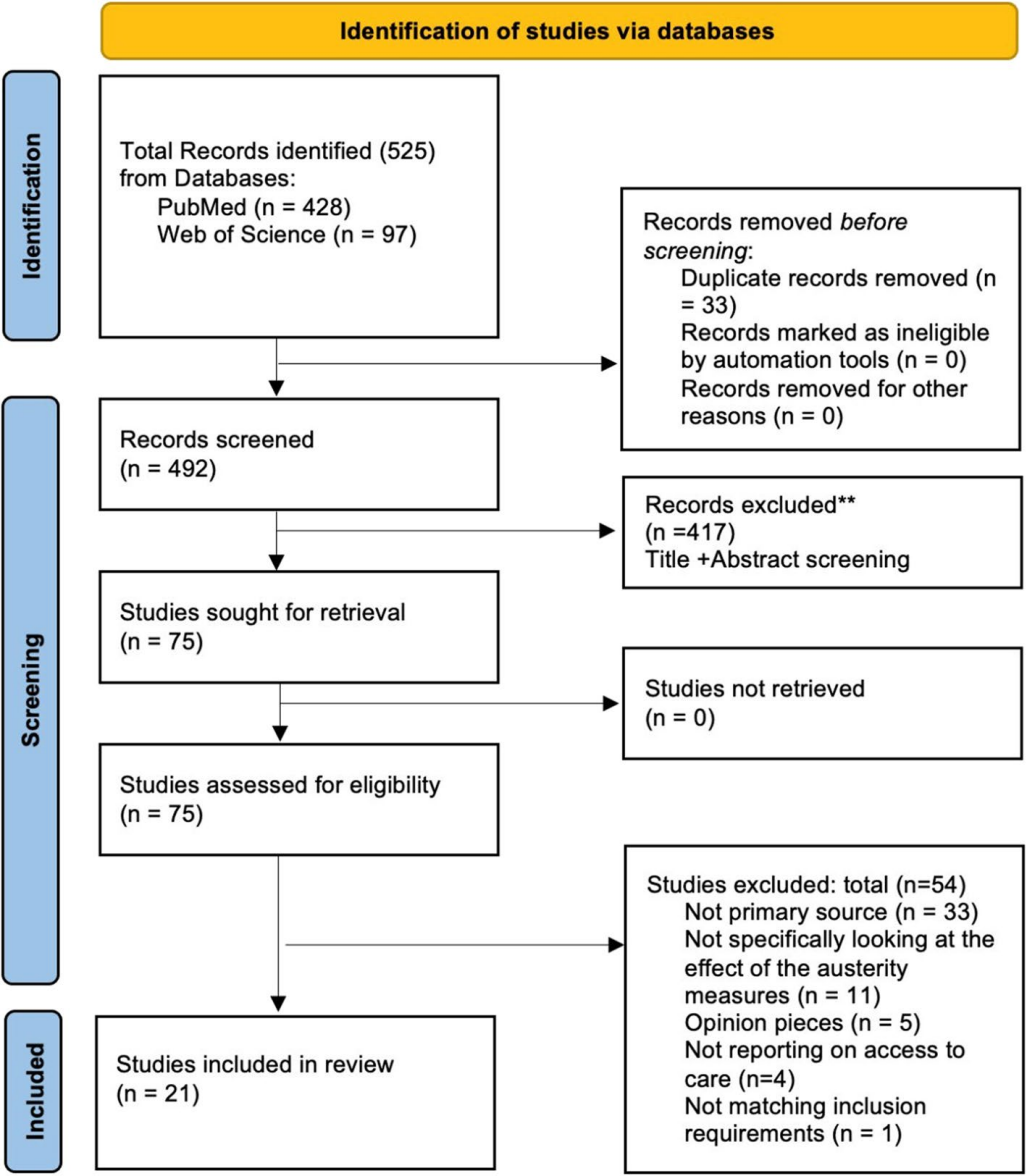
PRISMA flow diagram of the study selection and identification procedure

All results from the search were entered into Covidence [31] for screening. An Excel file with all results was downloaded to systematise the screening procedure. One author (CS) reviewed each title and abstract to exclude those that did not meet the inclusion criteria. Results were discussed with EP and JD. Uncertainties were resolved between the three authors. The full text of each selected article was reviewed independently by CS and discussed with JD and EP to determine whether they should be included in the data extraction phase. Any conflict resolution was handled by joint discussion (Fig. 1).

### Data charting process

CS developed the data extraction process, which was based on the previously defined research questions and certain general characteristics of the study. The final structure and organisation of the data extraction were discussed and decided upon with JD and EP. CS carried out data extraction with constant consultation with JD and EP in case of ambiguities.

### Data items

The following data elements were extracted from each publication (Table 1).

**Table 1:** Data items

Main data items	Description	Detailed process
**Authors and date of publication**	Extraction of authors and the date of publication.	–
**Study design**	Categorisation by primary studies of quantitative and qualitative nature.	–
**Time period and location researched**	Description of research period and research location (e.g., regions).	–
**Study description**	A free text description of the study	Description according to study aims
**Categorisation**	A free-text description of study outcomes categorised through thematic analysis into major themes	The categorisation process of the study outcomes related to specific barriers was based on the framework of Levesque et al. (2013) [23]. UMN emerged as one central theme during the categorisation process. UMN was not clustered under the categories of Levesque et al. (2013), as the concept of UMN concerns the aggregated barriers for measuring access to healthcare [23]. The concept of UMN was based on the EU-SILC survey allowing full comparability. The four major themes emerged as follows: 1) Unmet Healthcare Need; 2) Availability and Accommodation; 3) Affordability of Access to Healthcare; 4) Appropriateness
**Organisation:**	Free text descriptions of the four emerged main themes were broken down into two subthemes.	The following two recurring sub-themes emerged per major theme: “Austerity on the general population” and “Austerity on vulnerable population groups”. Vulnerable populations are defined as individuals that are disadvantaged in one or more socioeconomic factors (e.g., income, employment, housing and education) and may have difficulty accessing healthcare and receiving a certain quality of care which can affect individuals’ health. This state, in turn, causes them to be at higher risk for disparate healthcare access and outcomes [32].

### Critical appraisal of individual sources of evidence

No formal critical appraisal was applied.

### Synthesis of results

The above-described (Data items) recorded characteristics of the studies were recorded in a table (Supplementary material 4). For ease of orientation, each study included was given an ID number.

A full-text description of the recorded data according to the developed themes was carried out.

## Results

The final search strategy resulted in 525 articles. After excluding 33 duplicates, the remaining 492 articles were reviewed by title and abstract. Thus, 75 studies qualified for full-text analysis. After the full-text analysis, 21 studies were included in the study. Table 2 provides a list of the 21 included studies, each with an ID number, for simplicity. Table 2 is an extended version of Supplementary material 4 where a summary of all study characteristics can be found.

**Table 2:** Indexing table of the included studies

ID	Author (Publication Year)	Name of Study
1	Castano et al. (2016) [33]	Restricting access to healthcare to immigrants in Barcelona: A mixed-methods study with immigrants who have experienced an infectious disease
2	Cervero-Liceras et al. (2015) [34]	The effects of the financial crisis and austerity measures on the Spanish healthcare system: A qualitative analysis of health professionals’ perceptions in the region of Valencia
3	Córdoba-Doña et al. (2018) [35]	Withstanding austerity: Equity in health services utilisation in the first stage of the economic recession in Southern Spain
4	Dimitrovová & Perelman (2018) [36]	Changes in access to primary care in Europe and its patterning, 2007–12: a repeated cross-sectional study
5	Doetsch et al., (2017) [21]	Potential barriers in healthcare access of the elderly population influenced by the economic crisis and the troika agreement: a qualitative case study in Lisbon, Portugal
6	Gea-Sánchez et al. (2021) [37]	The resistance of nurses to austerity measures in the health sector during the financial crisis in Spain
7	Gogishvili et al. (2021) [38]	A qualitative study on mixed experiences of discrimination and healthcare access among HIV-positive immigrants in Spain
8	Heras-Mosteiro et al. (2016) [39]	Healthcare austerity measures in times of crisis: The perspectives of primary healthcare physicians in Madrid, Spain
9	Karanikolos et al. (2016) [40]	Access to care in the Baltic States: Did crisis have an impact?
10	Legido-Quigley et al. (2016) [41]	Effects of the financial crisis and Troika austerity measures on health and healthcare access in Portugal.
11	López-López et al. (2021) [42]	Catastrophic household expenditure associated with out-of-pocket healthcare payments in Spain
12	Petrelli et al. (2019) [43]	The geography and economics of forgoing medical examinations or therapeutic treatments in Italy during the economic crisis
13	Porthé et al. (2016) [44]	Changes in access to healthcare for immigrants in Catalonia during the economic crisis: Opinions of health professionals and immigrant users
14	Rachiotis et al. (2014) [45]	Medical supplies shortages and burnout among Greek healthcare workers during economic crisis: A pilot study
15	Rizzi et al. (2019) [46]	Older People Health and Access to Healthcare: A Retrospective look at Inequality Dynamics over the Past Decade
16	Rodríguez-Álvarez et al. (2019) [47]	Health Services Access Inequalities Between Native and Immigrant in a Southern European Region
17	Schneider & Devitt (2018) [48]	Accessing healthcare in times of economic growth and economic downturn: Evidence from Ireland
18	Souliotis et al. (2016) [49]	Access to care for multiple sclerosis in times of economic crisis in Greece – the hope ii study
19	Souliotis et al. (2014) [50]	Barriers to accessing biologic treatment for rheumatoid arthritis in Greece: The unseen impact of the fiscal crisis - The Health Outcomes Patient Environment (HOPE) study
20	Torfs et al. (2021) [12]	The unequal effects of austerity measures between income-groups on the access to healthcare: a quasi-experimental approach
21	Zavras et al. (2016) [51]	Economic crisis, austerity and unmet healthcare needs: the case of Greece

### Study characteristics of the included studies

The 21 studies are composed of the following study designs: longitudinal (*n* = 10), qualitative (n = 5), cross-sectional (*n* = 5) and time-series analysis (*n* = 1). The period covered ranges from 2003 (*n* = 3) until 2014/2015 (*n* = 4), though the majority stops at 2011/2012 (n = 7).

The year 2007/2008 was taken by most (n = 5) as the year when the crisis hit Europe. The variance of included studies between the different countries was very large: Spain (*n* = 9), Greece (n = 5), Ireland (n = 2), Italy (*n* = 2), Portugal (n = 2), Lithuania (n = 1), Latvia (n = 1), Estonia (n = 1), the United Kingdom (n = 1) and Europe as a whole (n = 1).

The studies indicated the main affected vulnerable populations groups as follows: patients with chronic diseases, elderly, (undocumented) migrants, unemployed, economically inactive and individuals with lower levels of education or socioeconomic status.

### Main findings

**Fig. 2:**
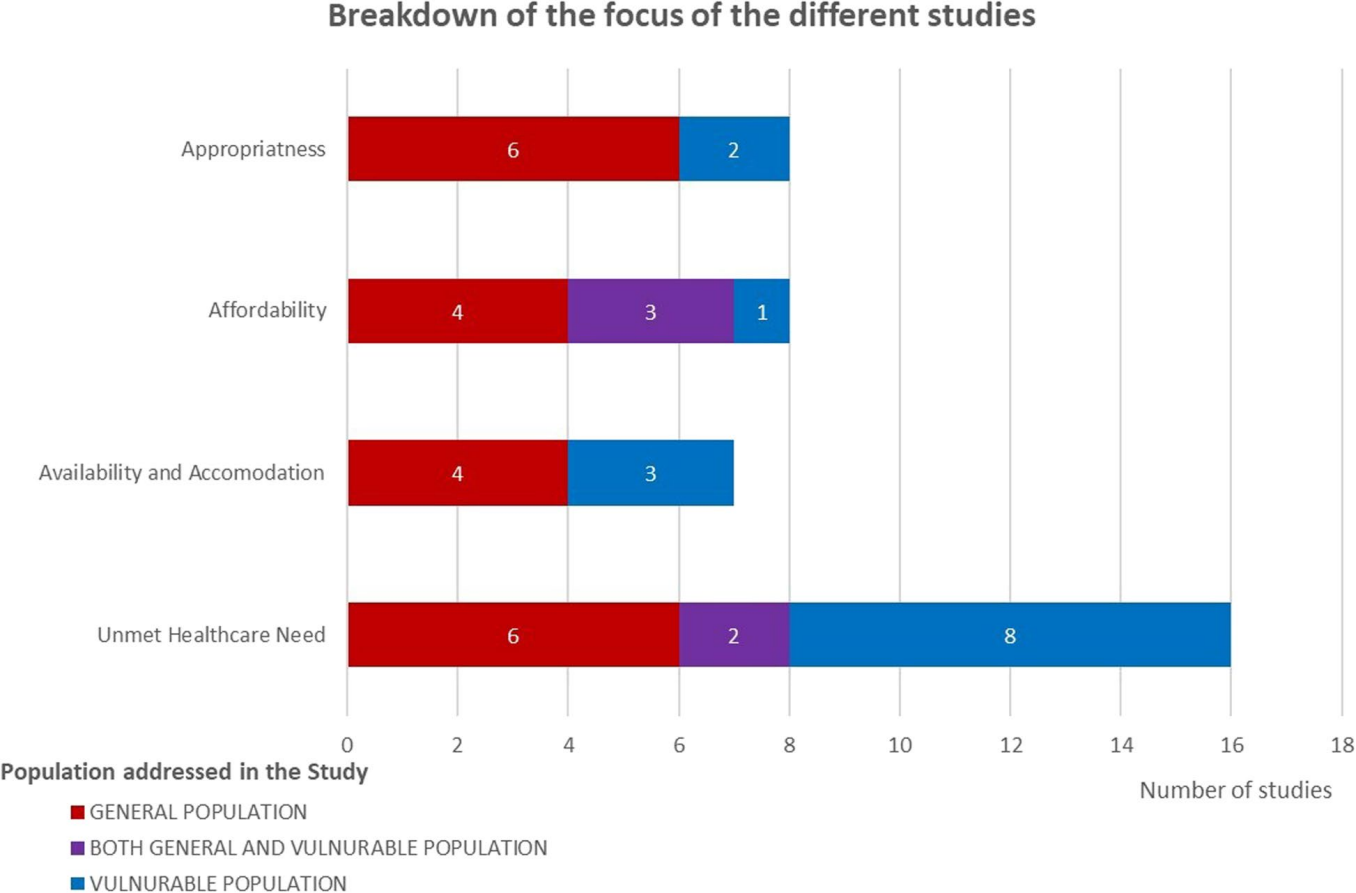
Number of studies broken down by central theme and the populations that were addressed

The results were clustered according to the four emerged themes, based on Levesque et al. (2013). First, the impact of austerity on the general population and then on the vulnerable population group was addressed. Keeping the theme format allowed to display a structured overview. The frequency of central themes and which populations were discussed is displayed in Fig. 2.

### (1) Unmet Healthcare Need

Results indicated that there was a general increase of UMN across multiple countries that applied austerity measures (e.g., Ireland, Estonia, Latvia, Italy, Greece, and Portugal), except for Lithuania, where no significant changes were found. UMN was mentioned by the majority (76%) of the studies.

#### General population

Six out of eight studies found an overall negative impact of austerity measures causing UMN and a decrease in the utilisation of healthcare. In Greece, there was already a significant increase in UMN between the periods 2004–2007 and then again in 2010–2011 (ID21). In Portugal, the rate of reporting UMN more than doubled from 2010 to 2011, which was the year in which the Memorandum of Understanding was implemented (ID10). Ireland had more significant increases in UMN than the UK, which maintained its healthcare spending trends (ID20). A study covering three Baltic countries (Latvia, Estonia and Lithuania) found adverse impact on UMN after the implementation of austerity measures (ID9). In Latvia, UMN rose from 15.4% in 2009 to 21% in 2011 and then fell back to 18.6% in 2012, however, without falling to pre-crisis levels (ID9). Compared to Latvia, Estonia faced substantially lower rates of UMN but was also found to have had a significant increase in UMN. In contrast to Latvia and Estonia, Lithuania experienced the lowest rates and stagnation in UMN, where cuts were mainly implemented by healthcare providers (ID9). In Italy, a slight increase in UMN and large regional differences were reported, with few changes in the Northern region, a moderate increase in the Centre region, and a high increase in the Southern region (ID12). In Ireland, an overall increase in UMN was associated with difficulties in accessing healthcare services after the crisis (49.3% in 2003; 52.8% in 2007; 62.3% in 2011) (ID17). In Andalusia, Spain, no decrease in the use of relevant health services was revealed (ID3). Although access to primary services across Europe was found to have increased between 2007 and 2012, it was found to be lower in countries that introduced austerity measures in healthcare (ID4).

#### Vulnerable population groups

In Portugal, UMN more than doubled after austerity was implemented in 2012 when compared to 2010: i) for the unemployed (OR 2.07; 95% CI 1.32–3.24); ii) for pensioners (OR 2.00; 95% CI 1.40–2.85); iii) and other economically inactive groups (OR 1.81; 95% CI 1.11–2.96), iv) for the employed it almost tripled (OR 2.82; 95% CI 2.15–3.69) (ID10). Vulnerable groups (e.g., unemployed and other economically inactive groups) were less affected as, at the same time, exemptions of co-payments were implemented (ID10). Italy also had a higher rate of UMN for individuals at risk of poverty, which increased over time (aOR = 1.54 in 2004–07; aOR = 1.70 in 2008–12, aOR = 2.21 in 2013–15) and for people with lower levels of education, foreign nationals, and those with chronic or severely limiting illnesses (ID12). Italy also presented high regional variance: though the northern region had no changes in the number of doctor visits, in the southern region, in which a higher proportion of individuals with a low economic status live, doctor visits fell by a third from 2006 to 2015 (ID15).

Seven (ID1, ID2, ID6, ID7, ID8, ID13, ID16) studies refer to the Spanish “Special Case of the Royal Decree-Law 16/2012” (RDL16\2012) that was implemented in line with austerity policy. The RDL16\2012 made it formally more difficult for immigrants to access the health card, a mandatory document to receive basic healthcare, yet the impact of this implementation was contradictory across studies. On the side of healthcare delivery, the restriction of insurance coverage specifically for undocumented immigrants and difficulties in obtaining a health card were emphasised by healthcare workers (ID13). Furthermore, 43% of caregivers answered that access to healthcare had decreased (ID6). However, in other studies, neither differences in healthcare use between native and foreign-born (ID16) nor denial of access to healthcare were reported (ID8). Two studies emphasised civil disobedience on the part of a proportion of healthcare professionals who did not implement the new restrictions introduced by RDL 16\2012 (ID2, ID6). On the side of immigrant healthcare users, loss of trust in the social system, anxiety, reduced use of primary care, increased use of emergency services, and sharing of healthcare cards were communicated (ID1). Furthermore, the majority of interviewed immigrants reported being at risk of losing access to the healthcare system (ID1) and faced restrictive insurance coverage (ID13) and issues in obtaining healthcare for HIV-positive immigrants (ID7).

### (2) Availability and Accommodation

Availability and accommodation were discussed in 33% (*n* = 7) of the included studies (ID2, ID5, ID6, ID9, ID17, ID18, ID19). They described similar barriers and reductions in access related to availability and accommodation. The prominent reasons were the distant geographical location of healthcare services, limited staff capacity, transport cuts to healthcare services, longer waiting lists for appointments, difficulties in scheduling appointments, and issues in promptly accommodating patients.

#### General population

In Spain, healthcare professionals perceived that access to care has decreased due to a reduction in the number of beds and the availability of out-of-hours emergency care in rural areas due to austerity measures (ID2). In Estonia, an increase in UMN due to austerity-related inaccessibility to health facilities was disclosed and partly attributed to the availability (e.g., distance) of health centres (ID9). In Ireland, though UMN based on having difficulties in reaching medical services decreased in 2007 and 2011 compared to 2003, these results were referred to the manner of reporting (one main reason allowed) (ID17). In Spain, austerity measures led to an increase in waiting times and waiting lists for procedures and consultations (ID6, ID2).

#### Vulnerable population groups

In Portugal, the availability of GPs and nurses declined due to reduced pension schemes and cuts in free non-emergency patient transport through austerity measures, causing barriers to accessing healthcare for the elderly (ID5). Another study in Portugal found that reaching a family doctor was challenging for individuals with chronic illnesses, making it difficult for them to receive their medicine (ID19). Patients in Greece with certain chronic diseases, such as multiple sclerosis or rheumatoid arthritis, have complained of appointment delays (ID18, ID19). Due to the austerity policy’s cost-cutting initiatives, they had increased difficulties getting their prescribed medications (ID18, ID19). The National Organization for Healthcare Services Provision pharmacies and specialists (e.g., rheumatologists) working in the public healthcare system were the only places that could prescribe and distribute some expensive medications as part of austerity measures (ID18, ID19).

### (3) Affordability to healthcare access

Affordability to access healthcare facilities was discussed in 38% (*n* = 8) of the included studies highlighting that unaffordability of health services was one of the critical consequences of austerity (ID2, ID5, ID8, ID9, ID10, ID17, ID21, ID11). Seven of the eight studies reported difficulties in accessing care related to affordability (ID 2, ID5, ID8, ID9, ID10, ID17, ID21).

#### General population

In Portugal, the likelihood of reporting financial difficulties in accessing care increased by about 70% between 2010 and 2012 (OR 1.68, 95% CI 1.32–2.12) (ID10). In Latvia, the general increase in UMN between 2010 and 2012 was found to have mainly derived due to the inability to afford healthcare (ID9). In Greece, UMN based on financial reasons were 44% higher in 2011 after the implementation of austerity measures when compared to 2006 (ID21). In Ireland, the number of patients reporting difficulties in covering the costs of medical treatment increased: from 39.2% in 2007 to 44.2% in 2011 (ID17). In Spain, health professionals expressed concerns about the introduction of co-payments through austerity for prescription drugs, as patients would not follow their care plan because of high costs (ID2, ID8). In contrast, another study in Spain did not find any change in the proportion of individuals who had catastrophic household expenses linked to out-of-pocket payments (ID11).

#### Vulnerable population groups

In Portugal, the increase in co-payments and adjustment of exemption schemes for the elderly and, in particular chronic patients was reported as one major reason to have made healthcare access more difficult as exemption schemes were not always perfectly tailored as co-morbidities were not included (ID5). Another study in Portugal concluded that the criteria for exemptions for certain conditions (e.g., chronic obstructive pulmonary disease, chronic active hepatitis) were tightened with austerity (ID10). Notably, in Ireland and Portugal, patients with the lowest incomes were not the most affected by austerity measures due to exemptions from co-payments for individuals on low incomes or in other precarious situations; instead, patients from a middle-income group were most likely to mention financial constraints as a reason for not meeting health needs (ID5, ID17). In Greece, patients from lower-income groups and the unemployed were more likely to cite financial reasons as the main cause of UMN when compared to the general population (ID21).

### (4) Appropriateness

Appropriateness of healthcare was discussed in 38% (*n* = 8) included studies (ID2, ID5, ID8, ID9, ID10, ID13, ID14, ID17). The main reason was limited access to high-quality health provision, which was described to be affected by limited staff availability, supply shortages, and long waiting times.

#### General population

In Spain, health professionals criticised the lack of basic items (e.g., sanitary pads) and reported that due to an austerity-related recruitment freeze, the remaining doctors had to divide the same amount of work between fewer doctors, which affected the quality of care (ID8, ID2). In Greece, a study revealed that 88% of respondents referred to supply shortages as a result of austerity measures, and 84% of those who reported supply shortages described that these shortages had a negative impact on the quality of care (ID14). In Estonia and Portugal, an increase in waiting times at the healthcare centre was reported to have amplified UMN (ID 9, ID10). In Portugal, the likelihood of reporting waiting times as a reason for UMN more than doubled (OR 2.18; 95% CI 1.20–3.98) after the implementation of austerity measures (ID10). In Estonia, a significant increase in UMN attributed to waiting times was described (ID9). In Ireland, waiting times at healthcare centres were described to have decreased (ID17).

#### Vulnerable population groups

In Portugal, the quality of care for older people was reported to have decreased as healthcare professionals had less time available, which affected their attitude towards patients and the appropriateness of care delivered (ID5). In Spain, cuts induced by austerity were reported to have resulted in higher cases of self-medication, increased emergency room visits, an increase in waiting times for an appointment with a GP leading to immigrants forgoing care, and a decrease in cultural mediators complicating the provision of appropriate care to migrants (ID13).

## Discussion

Results revealed that austerity policy had been largely associated with a reduction in access to healthcare across the EU-28 zone. This impact was mainly seen in the overall increase in rates of UMN and utilisation of healthcare and along the categories defined by Levesque et al. (2013), namely affordability, appropriateness and availability and accommodation [23]. Results revealed that when specific safeguards were not in place, such as the provision from Ireland enabling free healthcare for patients with a low income [48], vulnerable populations were more affected by the implemented austerity measures than the general population regarding their access to care.

The results of this review support other studies at the European general population level [52–54]. Despite substantial cross-country differences, results suggest that the interaction of fiscal austerity with economic and financial recessions and weak social protection may lead to a social crisis with a negative impact on healthcare access, especially for vulnerable populations [52]. European countries that were classified as having implemented higher levels of austerity, such as Greece, Spain, Portugal, and Ireland, reported a substantially greater deterioration in healthcare quality [24]. The variance and geographical variability of included studies in this analysis overlapped with those countries that implemented higher levels of austerity. This suggests that those countries, which implemented higher levels of austerity, also reported higher UMN [55–57].

Looking at the specific austerity measures using Malinovsky’s (2012) categorisation, no clear picture emerges as to whether some forms of austerity were less or more harmful than others. For example, it is not possible to determine whether a mere change in the financing mechanism, such as an increase in co-payments, was less harmful than the introduction of measures to reduce health care costs, such as wage cuts for health workers. Moreover, it is almost impossible to make these comparisons because austerity measures were often not limited to one category only [12]. Further evidence for the argument of the diffuse effects of austerity measures is provided by a review at the European general population level [56]. It stipulates two mechanisms that affect health in European countries that applied austerity: the indirect ‘social risk effect’ (e.g., through increasing unemployment) and the direct ‘healthcare effect’ (e.g., through cuts to healthcare services, restricting access to care) [56]. Both of these mechanisms were also argued to affect affordability in the included studies. Whereas Zavras et al. (2016) conclude that the rise in unaffordability of healthcare access was attributed to the crisis-related increase in unemployment in Greece, Karanikolos et al. (2016) attribute the increase in unaffordability to austerity measures in Latvia (introduction of co-payments) [40, 51]. Thus, large differences across studies can be seen, and the causal picture for UMN is not uniform.

All four emerged access categories reflect that the impact of austerity undermined access to healthcare, especially for vulnerable populations. This may have long-term negative consequences for health (e.g., worsening health status of patients with chronic conditions) and have adverse implications on the right to social security influencing the social security system [58]. Country-specific examples show that though generally negative consequences were reported in many countries, exemption provisions (e.g. free healthcare for lower-income groups) such as those in Ireland [48], if comprehensive enough, can mitigate the financial burden of the more vulnerable populations. In line with these results, Sakellariou and Rotarou (2017) argue with the example of Greece that austerity measures under neoliberal policy compromised appropriate access to care for individuals with disabilities by making the challenging attempt to maintain quality of care with fewer resources [22]. These findings contradict the claim often made by neoliberal advocates that a greater amount of good quality care can be provided with fewer resources [59]. It is noteworthy that, contrary to the Health in All Policies collaborative approach (HiAP) addressing health in policymaking across sectors, the European Commission did not assess what impact the austerity measures would have on health [60]. DG SANTE mainly gave advice on where possible cuts in health systems could be made instead of assessing the impact of these measures on the health of individuals in the member states [53].

As a result of the decrease in public health expenditure, austerity policy is argued to have only deepened the effects of the crisis [61]. In the same way, the particular case of the implementation of the “Special Case of the Royal Decree-Law 16/2012” (RDL16\2012) in Spain, mentioned in several of the included studies, can be also framed under the neoliberal policy scheme [62]. Legido-Quigley et al., (2013) [63] argued that the cuts enacted in RDL16\2012 to the Spanish healthcare system were not primarily aimed at reducing costs but are part of a larger neoliberal effort to reduce “the size of the state” [62]. This is a belief that stems from the idea of *the big state*, arguing that a state should take a smaller role in individual lives and that the individual, in conjunction with the private sector, can efficiently and effectively get what is needed and wanted [64]. Yet, a particular example of how austerity measures have led not to privatisation but to a greater role for the state was observed in Greece, which illustrates the diversity of austerity measures [64]. The enacted austerity measures stipulated that doctors with public contracts could only prescribe certain expensive drugs for patients with specific chronic diseases and dispensed only by public pharmacies in Greece. As a result, the access of certain regular patient groups was reduced because of geographic access barriers [49, 50]. Independently of the “size of the state”, the shift of the financial burden of healthcare from the state to the individual implemented through austerity impeded access to care. It affected the most vulnerable the most, as reflected in our results [48].

The adverse impacts of austerity on healthcare access, as the results of this study revealed, are in line with what is hypothesised when applying the TFC and the PEH theory [15, 17]. The developed hypothesis assumed that inequalities in health can derive from inequalities in access to resources, which in turn result from policy choices [18]. Another dimension of the PEH theory emphasises that the range of a person’s possible health status is limited by their situation in the social and economic system, which implies that the social status and material conditions of an individual have a significant impact on access. This reflects the differences found between the general population and vulnerable groups. Schrecker et al. (2019) argue that undermining the health system often hits the most vulnerable hardest, thus depriving them of the opportunity to reach their full health potential [65]. This highlights the link between the PEH theory and economic adjustment [65]. From an ethical perspective, it is deeply problematic that austerity appears to cause the most harm to the very sections of the population that are supposed to benefit from  the greatest protection – namely, the most vulnerable people in society.

IMF lending programs have impacted health equity, increased neonatal mortality, and reduced access to healthcare also at the international level [66]. An article published in the IMF’s journal Economy and Development revealed a 180-degree turnaround of the IMF by concluding that the “neoliberal agenda and austerity measures of recent years have done more harm than good” after reflecting on the human costs [67]. Though the head of the IMF communicated that “no one wants needless austerity”, the IMF continues to believe that it is a necessary tool that is unlikely to be dispensed with, particularly in the case of fiscally unbalanced countries [68]. In 2021, the managing director of the IMF communicated with respect to the current COVID-19 crisis that “Europe should be careful to not suffocate the newly found growth with introducing austerity measures” [69].

Thus, a rethinking of austerity seems to be taking place for the time being, as the EU has embarked on a new era of European deficit policy with its *Next Generation EU plan*, adopted in March 2021. The current COVID-19 crisis response plan, which is intended to take care of the accumulated debts of EU states, is a stark contrast to the structural adjustment programs established after the 2008 financial crisis, which were based, among other things, on cuts in the social and healthcare systems [70]. Yet, there are contradictory opinions on whether a period of austerity will return in light of the new inflationary pressures in conjunction with the increased debt caused by the COVID-19 crisis [71]. With the possibility that austerity is possibly being back on the political agenda, it is crucial for policymakers to find mitigating effects of austerity, especially on vulnerable population groups, in line with the collaborative approach of HiAP.

### Strengths and limitations

Strengths of this research include its comprehensive overview of studies across Europe that implemented austerity, which affected healthcare access. This research can be considered very timely as austerity may have a lag effect and often can only be fully understood in the post-crisis period. As the majority of studies referring to UMN are based on EU-SILC, it enables comparability between studies. The relevance of the study can be found in its lessons learned, which are linked to the COVID-19 crisis, demonstrating it to be a highly up-to-date topic.

The fact that most of the studies included are of ecological nature does not allow us to make definitive statements about austerity measures as the recorded change may have been biased by the product of another factor that is unknown (inability to control for confounding). Publication bias cannot be ruled out as the study selection was limited to studies published in English. Though study variance and geographic variability reflect the level of austerity implemented, as many countries were represented by only one study, there was little opportunity to validate and compare the findings with other studies in the respective countries. The great variance between the introduction of austerity measures made it harder to allow a comparison of the results, which may have affected the validity of the results.

## Conclusion

Results indicate that for several EU member states, the introduction of austerity measures caused decreased access to healthcare. The majority of studies reported a general increase in UMN, and issues in affordability, appropriateness and availability and accommodation of healthcare access. This study proved that vulnerable populations such as lower-income groups and immigrants were harder affected by many implemented austerity measures if their governments did not introduce some precautionary measures. The expected long-term consequences on health and the adverse implications on the right to social security require policy action. Based on the findings of this research, we propose policymakers at the national and international levels should evaluate the possible negative effects of implementing austerity measures on healthcare access and, if necessary, only impose them in conjunction with protective measures for the most vulnerable. Vulnerable populations should be harmed least, not most, by any change in health policy.

## Supplementary Information

**Additional file 1 **Supplementary material 1 Implemented policy measures as a response to the Great Recession in 2008. *This table summarises the policy responses to the 2008 financial crisis of some European countries in the field of healthcare.* Substituted table by Torfs et al. (2021) based on Mladovsky et al. (2012) AT Austria, CH Switzerland, CZ Czech Republic, DE Denmark, EE Estonia, ES Spain, FR France, GR Greece, IR Ireland, IS Iceland, LI Lithuania, LV Latvia, NL the Netherlands, PT Portugal, SI Slovenia, UK United Kingdom

**Additional file 2.** Supplementary material 2 Preferred Reporting Items for Systematic reviews and Meta-Analyses extension for Scoping Reviews (PRISMA-ScR) Checklist. This table indicates on which page the preferred reporting items can be found according to the PRISMA-ScR checklist. N/A

**Additional file 3.** Supplementary material 3 Search Strategy Outline. This table shows the search strategy divided into concepts, MeSH terms and keywords. The complete search strategy is also included. Acronyms - MeSH: Medical Subject Headings, TIAB: Title and Abstract

**Additional file 4.** Supplementary material 4. Characteristics of the selected studies. This table portrays the selected questions on healthcare quality by migrant mothers with respective response scales in the Migrant Friendly Maternal Care Questionnaire. AM = Austerity Measures; UMN: Unmet medical need; RDL: Royal Decree-Law 16/2012 (law restricting entitlement to care for unregistered migrants); PC: Primary care; GP: General practitioner; EMS: Emergency medical services; SE- status: Socioeconomic status; MS: Multiple sclerosis, RA: Rheumatoid arthritis. In the present table, the results for vulnerable groups and the general population have been summarised for reasons of limited available space

## Data Availability

All data generated or analysed during this study are included in this published article [and its supplementary information files].
