# Supplementary material for: A scoping review on the impact of austerity on healthcare access in the European Union: rethinking austerity for the most vulnerable
Source: Int J Equity Health. 2023 Jan 5;22:3. doi: 10.1186/s12939-022-01806-1 (PMC9815671; doi:10.1186/s12939-022-01806-1)
Supplement: Supplementary file 1 — Additional file 1 Supplementary material 1 Implemented policy measures as a response to the Great Recession in 2008. This table summarises the policy responses to the 2008 financial crisis of some European countries in the field of healthcare. Substituted table by Torfs et al. (2021) based on Mladovsky et al. (2012) AT Austria, CH Switzerland, CZ Czech Republic, DE Denmark, EE Estonia, ES Spain, FR France, GR Greece, IR Ireland, IS Iceland, LI Lithuania, LV Latvia, NL the Netherlands, PT Portugal, SI Slovenia, UK United Kingdom [file 12939_2022_1806_MOESM1_ESM.docx]

| Supplementary material 1: Implemented policy measures as a response to the Great Recession in 2008 | |
| --- | --- |
| Policy measure | Country |
| **Changing the level of contributions for publicly financed healthcare** | |
| Cutbacks | IS, IR, IT, GR, PT, ES |
| Increasing employee contribution rates | GR, PT, SI |
| Increasing or introducing user charges | IR, CH, CZ, DE, ES, FR, GR, IT, LV, NL, PT, SI |
| Expanding benefits, targeting low-income groups | IR, AT, FR, IT |
|  |  |
| **Affecting the volume and quality of publicly financed healthcare** | |
| Changing the scope of coverage | IR, EE, NL, PT, IR |
| Changing in the population of coverage | IR, ES |
| **Affecting the costs of publicly financed healthcare** | |
| Reducing the salaries of health professionals | IS, IR, UK, DE, FR, GR, LI, PT, SI |
| Changing in provider infrastructure and capital investment | IS, IR, DE, GR, LI, LV, NL, PT, SI |
| Centralization of the healthcare organizations (merging hospitals) | IS, DE, GR, LV, PT, SI |
| Reducing the tariffs paid to providers | IR, EE, SI |
| Source: Substituted table by Torfs et al. (2021) based on Mladovsky et al. (2012) AT Austria, CH Switzerland, CZ Czech Republic, DE Denmark, EE Estonia, ES Spain, FR France, GR Greece, IR Ireland, IS Iceland, LI Lithuania, LV Latvia, NL the Netherlands, PT Portugal, SI Slovenia, UK United Kingdom | |
|  | |
|  | |
|  | |
|  | |
