# Supplementary material for: A scoping review on the impact of austerity on healthcare access in the European Union: rethinking austerity for the most vulnerable
Source: Int J Equity Health. 2023 Jan 5;22:3. doi: 10.1186/s12939-022-01806-1 (PMC9815671; doi:10.1186/s12939-022-01806-1)
Supplement: Supplementary file 3 — Additional file 3. Supplementary material 3 Search Strategy Outline. This table shows the search strategy divided into concepts, MeSH terms and keywords. The complete search strategy is also included. Acronyms - MeSH: Medical Subject Headings, TIAB: Title and Abstract [file 12939_2022_1806_MOESM3_ESM.docx]

**Supplementary material 3. Search Strategy Outline**

| \| *Search strategy divided by concepts, MeSH terms and keywords* \| \| \| --- \| --- \| \| **Concept 1** \| Adults in European countries 🡪 Europe \| \| MeSH terms \| Europe [MeSH \| \| Keywords \| Eurozone[TIAB] OR Italy[TIAB] OR Spain[TIAB] OR Belgium[TIAB] OR France[TIAB] OR Germany[TIAB] OR Luxembourg [TIAB] OR Netherlands[TIAB] OR Denmark[TIAB] OR Ireland [TIAB] OR United Kingdom[TIAB] OR U.K.[TIAB] OR Greece[TIAB] OR Portugal [TIAB] OR [TIAB] OR Austria[TIAB] OR Finland[TIAB] OR Sweden[TIAB] OR Cyprus[TIAB] OR Czechia[TIAB] OR Estonia[TIAB] OR Hungary[TIAB] OR Latvia[TIAB] OR Lithuania[TIAB] Malta[TIAB] OR Poland[TIAB] OR Slovakia[TIAB] OR Slovenia[TIAB] OR Bulgaria[TIAB] OR Romania[TIAB] OR Croatia[TIAB] \| \| **Concept 2** \| Austerity \| \| MeSH terms \| "Economic Recession"[Mesh] OR "Cost Control"[Mesh] \| \| Keywords \| "Austerity measures"[TIAB] OR "Austerity-measures" [TIAB] OR "Financial crisis"[TIAB] OR "2008 financial crisis"[TIAB] OR "2008 Financial crisis" [TIAB] Economic adjustment program EAP Memorandum of Understanding MoU \| \| **Concept 3** \| Access to care \| \| MeSH terms \| "Health Services Accessibility"[Mesh] OR "Healthcare Disparities"[Mesh] OR "Health Care Rationing/economics"[Mesh] \| \| Keywords \| "Health Services Accessibility"[TIAB] OR "Access to care"[TIAB] OR Access[TIAB] OR Accessibility [TIAB]" \| \| Acronyms - MeSH: Medical Subject Headings, TIAB: Title and Abstract \| \|   **MESH terms**  (Europe [MeSH Terms] OR Eurozone[TIAB] OR Italy[TIAB] OR Spain[TIAB] OR Belgium[TIAB] OR France[TIAB] OR Germany[TIAB] OR Luxembourg [TIAB] OR Netherlands[TIAB] OR Denmark[TIAB] OR Ireland [TIAB] OR United Kingdom[TIAB] OR Greece[TIAB] OR Portugal [TIAB] OR Austria[TIAB] OR Finland[TIAB] OR Sweden[TIAB] OR Cyprus[TIAB] OR Czechia[TIAB] OR Estonia[TIAB] OR Hungary[TIAB] OR Latvia[TIAB] OR Lithuania[TIAB] OR Malta[TIAB] OR Poland[TIAB] OR Slovakia[TIAB] OR Slovenia[TIAB] OR Bulgaria[TIAB] OR Romania[TIAB] OR Croatia[TIAB]**) AND** ("Economic Recession"[Mesh] OR "Cost Control"[Mesh]OR Austerity[TIAB] OR "Austerity measures"[TIAB] OR "Austerity-measures"[TIAB] OR "austerity-measures"[TIAB]OR "austerity measures"[TIAB] OR "Austerity Measures"[TIAB] OR "Austerity-Measures"[TIAB] OR "Financial crisis"[TIAB] OR "2008 financial crisis"[TIAB] OR “Great recession”[TIAB]OR "2008 Financial crisis" [TIAB] OR “Memorandum of Understanding” [TIAB] OR “memorandum of understanding” [TIAB] OR “Memorandum-of-Understanding” [TIAB] OR MoU[TIAB] OR “Economic Adjustment Programme” [TIAB] OR EAP[TIAB] OR TROIKA[TIAB]**) AND** ("Health Services Accessibility"[Mesh] OR "Healthcare Disparities"[Mesh] OR "Health Care Rationing/economics"[Mesh]OR "Delivery of Health Care/economics"[MAJR] OR "Health Services Accessibility"[TIAB] OR "Access to care"[TIAB]) |
| --- | --- | --- | --- | --- | --- | --- | --- | --- | --- | --- | --- | --- | --- | --- | --- | --- | --- | --- | --- | --- | --- | --- |
