# Supplementary material for: A scoping review on the impact of austerity on healthcare access in the European Union: rethinking austerity for the most vulnerable
Source: Int J Equity Health. 2023 Jan 5;22:3. doi: 10.1186/s12939-022-01806-1 (PMC9815671; doi:10.1186/s12939-022-01806-1)
Supplement: Supplementary file 4 — Additional file 4. Supplementary material 4. Characteristics of the selected studies. This table portrays the selected questions on healthcare quality by migrant mothers with respective response scales in the Migrant Friendly Maternal Care Questionnaire. AM = Austerity Measures; UMN: Unmet medical need; RDL: Royal Decree-Law 16/2012 (law restricting entitlement to care for unregistered migrants); PC: Primary care; GP: General practitioner; EMS: Emergency medical services; SE- status: Socioeconomic status; MS: Multiple sclerosis, RA: Rheumatoid arthritis. In the present table, the results for vulnerable groups and the general population have been summarised for reasons of limited available space [file 12939_2022_1806_MOESM4_ESM.docx]

| **Supplementary material 4:** *Characteristics of the selected studies* | | | | | | | | |
| --- | --- | --- | --- | --- | --- | --- | --- | --- |
| ID | **Author (Year Publication)** | Study Design | Research period & Place | Topic/ Aim | Unmet health care needs  And general health care access | Access dimensions by Levesque et al. (2013) | | |
|  |  |  |  |  |  | **Availability & Accommodation** | **Affordability** | **Appropriateness** |
| **1** | Castano et al. (2016) | Cross-sectional | Barcelona - Spain, Summer 2012 | Mapping whether foreign-born persons with an infectious disease in Barcelona encountered problems accessing health care after enacting austerity measures (AM*). | Up to 66% of the immigrant respondents were found to be at risk of losing access to care services because of the introduction of the RDL* law |  |  |  |
| **2** | Cervero-Liceras et al. (2015) | Qualitative | Valencia - Spain, May 2013 - June 2013 | Health care professionals’ perception of the effects of austerity measures | Health care professionals opposed RDL* restricting access for migrants but had doubts if it was being implemented | Increase in Waiting lists and increase in difficulty to transfer patients to more appropriate facilities, closing of emergency units | Patients being unable to afford care due to co-payments | Decrease in quality of care due to reduced staff and limited recourses |
| **3** | Córdoba-Doña et al. (2018) | Longitudinal | Andalusia - Spain, 2007, 2011, 2012 | Describing trends in health care utilisation in horizontal inequity in the use of health services during the early years of the Great Recession | The study found no change in health care utilisation inequity after the implementation of AM* |  |  |  |
| **4** | Dimitrovová & Perelman (2018) | Longitudinal | Europe, 2007, 2012 | Analysing the change in access to PC* and its patterning in 28 European countries between 2007 and 2012 | Access to PC* improved between 2007 and 2012; improvement was greater in countries with higher health investments. Low-SE* status people report poorer access to PC*, and this was stable between 2007 / 2012 |  |  |  |
| * AM = Austerity Measures; UMN: Unmet medical need; RDL: Royal Decree-Law 16/2012 (law restricting entitlement to care for unregistered migrants); PC: Primary care; GP: General practitioner; EMS: Emergency medical services; SE- status: Socioeconomic status; MS: Multiple sclerosis, RA: Rheumatoid arthritis | | | | | | | | |

| *Characteristics of the selected studies* | | | | | | | | |
| --- | --- | --- | --- | --- | --- | --- | --- | --- |
| ID | **Author (Year Publication)** | Study Design | Research period & Place | Topic/ Aim | Unmet health care needs  And general health care access | Access dimensions by Levesque et al. (2013) | | |
|  |  |  |  |  |  | **Availability & Accommodation** | **Affordability** | **Appropriateness** |
| **5** | Doetsch et al., (2017) | Qualitative | Lisbon - Portugal, May - July 2016 | Potential barriers among the elderly population to healthcare access -influenced by the economic crisis and the troika agreement |  | The availability of GPs and nurses & the cuts of the free-of-charge non-emergency patient transportation were indicated to be a barrier to access for elderly. | Change in exemption allowance, co-pay being a barrier accessing care for elderly. Elderly of with a medium income were most affected | Attitude of care providers caring for the elderly was negatively affected |
| **6** | Gea-Sánchez et al. (2021) | Cross-sectional | Spain, 2013 | Nurses’ perspectives on how the financial crisis and austerity measures introduced affected their ability to provide care | Nurses mentioned a decrease in access for Migrants due to a lack of health cards | Nurses mentioned an increase in waiting list time due to the AM* |  | Nurses mentioned a decrease in quality of care |
| **7** | Gogishvili et al. (2021) | Qualitative | Valencia - Spain, Summer 2019 | Reporting on mixed experiences of discrimination and healthcare access among HIV*-positive immigrants in Spain after a change in regulation | A minority of the respondents (Migrants) noted not being able to receive a health card as a barrier when accessing care |  |  |  |
| * AM = Austerity Measures; UMN: Unmet medical need; RDL: Royal Decree-Law 16/2012 (law restricting entitlement to care for unregistered migrants); PC: Primary care; GP: General practitioner; EMS: Emergency medical services; SE- status: Socioeconomic status; MS: Multiple sclerosis, RA: Rheumatoid arthritis | | | | | | | | |

| *Characteristics of the selected studies* | | | | | | | | |
| --- | --- | --- | --- | --- | --- | --- | --- | --- |
| ID | **Author (Year Publication)** | Study Design | Research period & Place | Topic/ Aim | Unmet health care needs  And general health care access | Access dimensions by Levesque et al. (2013) | | |
|  |  |  |  |  |  | **Availability & Accommodation** | **Affordability** | **Appropriateness** |
| **8** | Heras-Mosteiro et al. (2016) | Qualitative | Madrid - Spain, Dec 2013 - Mar. 2014 | The effects of austerity measures implemented in the public health care system and their potential impacts on access and utilisation of primary health care services in the eyes of primary health care physicians | All participants indicated that despite the RDL* they would continue to administer care to migrants and would not deny access to health to anyone |  | Adverse effects of co-payment for prescription drugs and some cases of non-adherence to treatment were reported, with patients choosing to take less medication for financial reasons. | Nurses reported the hiring freeze affecting the quality of care because of a decreased nursing ratio |
| **9** | Karanikolos et al. (2016) | Longitudinal | Estonia, Latvia, Lithuania, 2005 - 2012 | Analysis of the impact of the economic crisis and post-crisis austerity measures on health systems and access to medical services in the three countries. | Unmet medical need (UMN*) increased both in Latvia and Estonia after the use of AM*, Lithuania being faced with way looser AM* did not experience a change in UMN* | In Estonia, the increase in UMN* can be traced back, among other things, to an increase in difficulties in reaching medical centres | In Latvia, the increase in unmet medical need was mainly rooted in an inability to afford care | In Estonia, the increase in UMN* can be traced back, among other things, to an increase in waiting times |
| * AM = Austerity Measures; UMN: Unmet medical need; RDL: Royal Decree-Law 16/2012 (law restricting entitlement to care for unregistered migrants); PC: Primary care; GP: General practitioner; EMS: Emergency medical services; SE- status: Socioeconomic status; MS: Multiple sclerosis, RA: Rheumatoid arthritis | | | | | | | | |

| *Characteristics of the selected studies* | | | | | | | | |
| --- | --- | --- | --- | --- | --- | --- | --- | --- |
| ID | **Author (Year Publication)** | Study Design | Research period & Place | Topic/ Aim | Unmet health care needs  And general health care access | Access dimensions by Levesque et al. (2013) | | |
|  |  |  |  |  |  | **Availability & Accommodation** | **Affordability** | **Appropriateness** |
| **10** | Legido-Quigley et al. (2016) | Longitudinal | Portugal, 2010, 2012 | Unmet medical need during the recession and before and after the Troika austerity package | The odds of reporting UMN* more than doubled in the year where AM had been introduced (2010 vs 2012) UMN* doubled for the unemployed, pensioners and other economically inactive groups - an even larger increase was observed among the employed |  | The odds of citing financial barriers increased by almost 70%. People with chronic diseases also mentioned increased difficulties in accessing their medicines due to co-payments. | The odds of citing waiting times as a barrier more than doubled |
| **11** | López-López et al. (2021) | Longitudinal | Spain, 2008 - 2015 | Analysis of the incidence and intensity of financial catastrophism derived from Spanish households' out-of-pocket payments related to health care |  |  | Catastrophic health expenditure related to out-of-pocket spending did not change significantly over the analysed time |  |
| * AM = Austerity Measures; UMN: Unmet medical need; RDL: Royal Decree-Law 16/2012 (law restricting entitlement to care for unregistered migrants); PC: Primary care; GP: General practitioner; EMS: Emergency medical services; SE- status: Socioeconomic status; MS: Multiple sclerosis, RA: Rheumatoid arthritis | | | | | | | | |

| *Characteristics of the selected studies* | | | | | | | | |
| --- | --- | --- | --- | --- | --- | --- | --- | --- |
| ID | **Author (Year Publication)** | Study Design | Research period & Place | Topic/ Aim | Unmet health care needs  And general health care access | Access dimensions by Levesque et al. (2013) | | |
|  |  |  |  |  |  | **Availability & Accommodation** | **Affordability** | **Appropriateness** |
| **13** | Porthé et al. (2016) | Qualitative | Catalonia - Spain, Nov. 2014 - Sep. 2015 | Analysing changes in immigrants’ access to health care during the economic crisis from the perspective of health professionals (medical and administrative) and immigrants | Users noted that obtaining a health card was made more difficult not only by increased requirements but also by stricter enforcement of practices |  |  | An increase in waiting times discouraged immigrants from seeking care and turned them to self-medication due to fear of losing employment.  Mediators were cut, making the provision of high-quality care for migrants difficult |
| **14** | Rachiotis et al. (2014) | Cross-sectional | Greece, April 2013 | Researching the frequency of medical supply shortages in two Greek public hospital departments and assessing their possible impact on the risk of burnout among healthcare workers |  |  |  | Among the participants, 88% reported significant healthcare bottlenecks in the last 12 months. In addition, 84% of participants who had experienced healthcare supply bottlenecks reported that these shortages had a negative impact on the quality of care |
| * AM = Austerity Measures; UMN: Unmet medical need; RDL: Royal Decree-Law 16/2012 (law restricting entitlement to care for unregistered migrants); PC: Primary care; GP: General practitioner; EMS: Emergency medical services; SE- status: Socioeconomic status; MS: Multiple sclerosis, RA: Rheumatoid arthritis | | | | | | | | |

| *Characteristics of the selected studies* | | | | | | | | |
| --- | --- | --- | --- | --- | --- | --- | --- | --- |
| ID | **Author (Year Publication)** | Study Design | Research period & Place | Topic/ Aim | Unmet health care needs  And general health care access | Access dimensions by Levesque et al. (2013) | | |
|  |  |  |  |  |  | **Availability & Accommodation** | **Affordability** | **Appropriateness** |
| **15** | Rizzi et al. (2019) | Longitudinal | Italy, 2006, 2007, 2011, 2013, 2015 | Assessing the evolution of inequalities in older people's health and access to healthcare in Italian macro areas since the beginning of the 2008 crisis | Doctor visits fell by a third of their pre-crisis level in the south of Italy. General higher GP* use in the south compared to the north for poorer older people while reducing visits to specialists |  |  |  |
| **16** | Rodríguez-Álvarez et al. (2019) | Longitudinal | Basque - Spain, 2010, 2012 | Investigating inequalities in access to different levels of health care according to the place of birth because of austerity measures | Both male and female migrants were using GP* and EMS* services more often than the general population. There was no difference for specialised care | No impact of the RDL* on limiting the access to care was found, mainly due to the region's resistance to compliance |  |  |
| **17** | Schneider & Devitt (2018) | Longitudinal | Ireland, 2003, 2007, 2011 | Examines the accessibility of healthcare in Ireland between 2003 and 2011 in the context of strong economic growth (2003–2007) and the subsequent financial crisis, which began in 2008 | Difficulties in accessing health care already increased from 2003 to 2007; this trend continued to 2011 | Difficulties related to physically reaching medical treatment and scheduling an appointment decreased during the researched period | Difficulties in financing medical care increased from 2003 to 2011, but the most significant increase happened before the crisis. The lowest income group did not experience any changes in being able to afford care |  |
| * AM = Austerity Measures; UMN: Unmet medical need; RDL: Royal Decree-Law 16/2012 (law restricting entitlement to care for unregistered migrants); PC: Primary care; GP: General practitioner; EMS: Emergency medical services; SE- status: Socioeconomic status; MS: Multiple sclerosis, RA: Rheumatoid arthritis | | | | | | | | |

| *Characteristics of the selected studies* | | | | | | | | | | |
| --- | --- | --- | --- | --- | --- | --- | --- | --- | --- | --- |
| ID | **Author (Year Publication)** | | Study Design | | Research period & Place | Topic/ Aim | Unmet health care needs  And general health care access | Access dimensions by Levesque et al. (2013) | | |
|  |  |  |  |  |  |  |  | **Availability & Accommodation** | **Affordability** | **Appropriateness** |
| **18** | Souliotis et al. (2016) | | Cross-sectional | Greece, Nov. - Dec. 2012 | | Assessing the barriers to access rheumatoid arthritis (RA) treatment by recording patients’, rheumatologists’ and EOPYY* pharmacists’ experiences. |  | As reason for reduced accessibility to RA* medical treatment and pharmaceuticals, difficulties in reaching the pharmacies and prescribing doctors and delays in scheduling an appointment with the doctor were mentioned |  |  |
| **19** | Souliotis et al. (2014) | | Cross-sectional | | Greece, Jan. - Feb. 2014 | Assessing the barriers to Multiple sclerosis (MS*) medication after a change in the distribution system by recording MS* patients’ experiences. |  | As reason for reduced accessibility to MS* medical treatment and pharmaceuticals travelling difficulties and delays in scheduling an appointment with the doctor were mentioned |  |  |
| * AM = Austerity Measures; UMN: Unmet medical need; RDL: Royal Decree-Law 16/2012 (law restricting entitlement to care for unregistered migrants); PC: Primary care; GP: General practitioner; EMS: Emergency medical services; SE- status: Socioeconomic status; MS: Multiple sclerosis, RA: Rheumatoid arthritis | | | | | | | | | | |
|  | | | | | | | | | | |
| Characteristics of the selected studies | | | | | | | | | | |
| ID | **Author (Year Publication)** | | Study Design | | Research period & Place | Topic/ Aim | Unmet health care needs  And general health care access | Access dimensions by Levesque et al. (2013) | | |
|  |  |  |  |  |  |  |  | **Availability & Accommodation** | **Affordability** | **Appropriateness** |
| **20** | | Torfs et al. (2021) | Longitudinal | | Ireland, United Kingdom, Iceland*, Sweden* 2008, 2014 | This study examines the unequal impact of cuts in health spending on access to care for different income groups in European countries | In Ireland (higher AM*), the UNM* increased more compared to the United Kingdom  In Ireland, people with medium income were most affected |  | Protections implemented in Ireland for the lowest income group were effective |  |
| **21** | Zavras et al. (2016) | | Time-series analysis | | Greece, 2004 - 2011 | Assessing how economic crisis and austerity affected unmet healthcare needs in Greece | Significant increase in unmet health- care needs after the implemented austerity measures in Greece |  | The odds of naming financial as a reason for UMN increased by 44% in 2011 compared to 2006. Lower-income groups and the unemployed were even more affected |  |
| *  Results relating to Iceland being compared to Sweden were not included because the scope of the study covered only EU28 countries  AM = Austerity Measures; UMN: Unmet medical need; RDL: Royal Decree-Law 16/2012 (law restricting entitlement to care for unregistered migrants); PC: Primary care; GP: General practitioner; EMS: Emergency medical services; SE- status: Socioeconomic status; MS: Multiple sclerosis, RA: Rheumatoid arthritis  In the present table, the results for vulnerable groups and the general population have been summarised for reasons of limited available space. | | | | | | | | | | |
|  | | | | | | | | | | |
